# Long-term outcomes of CNS WHO grade 2 oligodendroglioma in adult patients: a single-institution experience

**DOI:** 10.1007/s12672-024-01136-4

**Published:** 2024-07-06

**Authors:** Yukyeng Byeon, Chaejin Lee, Juhee Jeon, Gung Ju Kim, Sangjoon Chong, Young-Hoon Kim, Young Hyun Cho, Seok Ho Hong, Chang-Ki Hong, Jeong Hoon Kim, Sang Woo Song

**Affiliations:** 1 Department of Neurological Surgery, Asan Medical Center, University of Ulsan College of Medicine, 88 Olympic-ro 43-gil, Songpa-gu, Seoul, 05505 Republic of Korea; 2 Department of Neurosurgery, Kyungpook National University Hospital, Daegu, 41944 South Korea

**Keywords:** Oligodendroglioma, 1p/19q codeletion, Extent of resection, Observation, Survival

## Abstract

**Purpose:**

Oligodendrogliomas (ODGs) are a subtype of diffuse lower-grade gliomas with overall survival of > 10 years. This study aims to analyze long-term outcomes and identify prognostic factors in patients with WHO grade 2 ODG.

**Methods:**

We retrospectively reviewed 138 adult patients diagnosed with 1p/19q co-deleted ODG who underwent surgical resection or biopsy between 1994 and 2021, analyzing clinical data, treatment details, and outcomes. Progression-free survival (PFS) and overall survival (OS) were evaluated using Kaplan–Meier analysis. Univariate and multivariate Cox regression analyses were utilized to identify significant prognostic factors.

**Results:**

In the gross total resection (GTR) group, 63 (45.7%) underwent observation and 5 (3.6%) received postoperative treatment; in the non-GTR group, 37 (26.8%) were observed and 33 (23.9%) received postoperative treatment. The median PFS and OS were 6.8 and 18.4 years, respectively. Between adjuvant treatment and observation, there was no significant difference in PFS or OS. However, GTR or STR with less than 10% residual tumor exhibited significantly better PFS and OS compared to PR or biopsy (p = 0.022 and 0.032, respectively). Multivariate analysis revealed that contrast enhancement on MRI was associated with worse PFS (HR = 2.36, p < 0.001) and OS (HR = 5.89, p = 0.001). And the presence of seizures at presentation was associated with improved OS (HR = 0.28, p = 0.006).

**Conclusion:**

This study underscores favorable long-term outcomes for patients with 1p/19q co-deleted ODG WHO grade 2. Our findings indicate that the EOR plays a crucial role as a significant prognostic factor in enhancing PFS and OS outcomes in WHO grade 2 ODG.

**Supplementary Information:**

The online version contains supplementary material available at 10.1007/s12672-024-01136-4.

## Introduction

Diffuse lower grade gliomas (LGGs) constitute a minority of all primary brain tumors, with oligodendrogliomas (ODGs) being particularly rare [1]. The reported overall incidence rate of ODG is 0.1 per 100,000 person-years in Korea and 0.23 in the USA [2, 3]. The 2021 World Health Organization (WHO) Classification of Tumors of the Central Nervous System (CNS) has refined the molecular definition of ODGs, emphasizing the importance of isocitrate dehydrogenase (IDH)-mutant status accompanied by 1p/19q co-deletion [4]. Recent data suggests a declining trend in ODG incidence, which may be attributed to changes in diagnostic criteria and the incorporation of molecular diagnostics [5].

ODGs, commonly presenting with seizures in adults, are predominantly located in the frontal lobe and are characterized by their slow growth. Upon categorizing patients based on their molecular subtype, it was observed that individuals diagnosed with ODG exhibit a longer overall survival compared to those with IDH-mutant astrocytomas [6].

Significant clinical trials, such as RTOG 9802, RTOG 9402, and EORTC 26951 have shown that the addition of PCV (procarbazine, lomustine, and vincristine) to radiotherapy (RTx) significantly improves overall survival (OS) and progression-free survival (PFS) in patients with ODG [7–10]. Following these trials, both the ASCO-SNO and NCCN guidelines now recommend the use of standard RTx with adjuvant PCV chemotherapy for WHO grade 2 ODG patients with high risk of tumor progression [11, 12].

Despite the availability of various treatment options, management of ODG remains highly heterogenous, often influenced by institutional practices and individual circumstances. Our study aims to bridge the gap in research regarding the long-term outcomes of WHO grade 2 ODGs, which are rare and typically manifest early in the disease course. Accordingly, this study focuses on analyzing long-term outcomes, including PFS, OS, and prognostic factors, in patients with WHO grade 2 ODG, featuring a well-defined molecular profile.

## Methods

### Study design

We conducted a retrospective study involving 271 patients diagnosed with histological oligoastrocytoma and ODG who underwent surgical resection or biopsy at a single institution between January 1994 and December 2021. Aligning with the 2021 WHO CNS tumor classification, we reclassified all patient cases to molecular diagnosis.

**Fig. 1:**
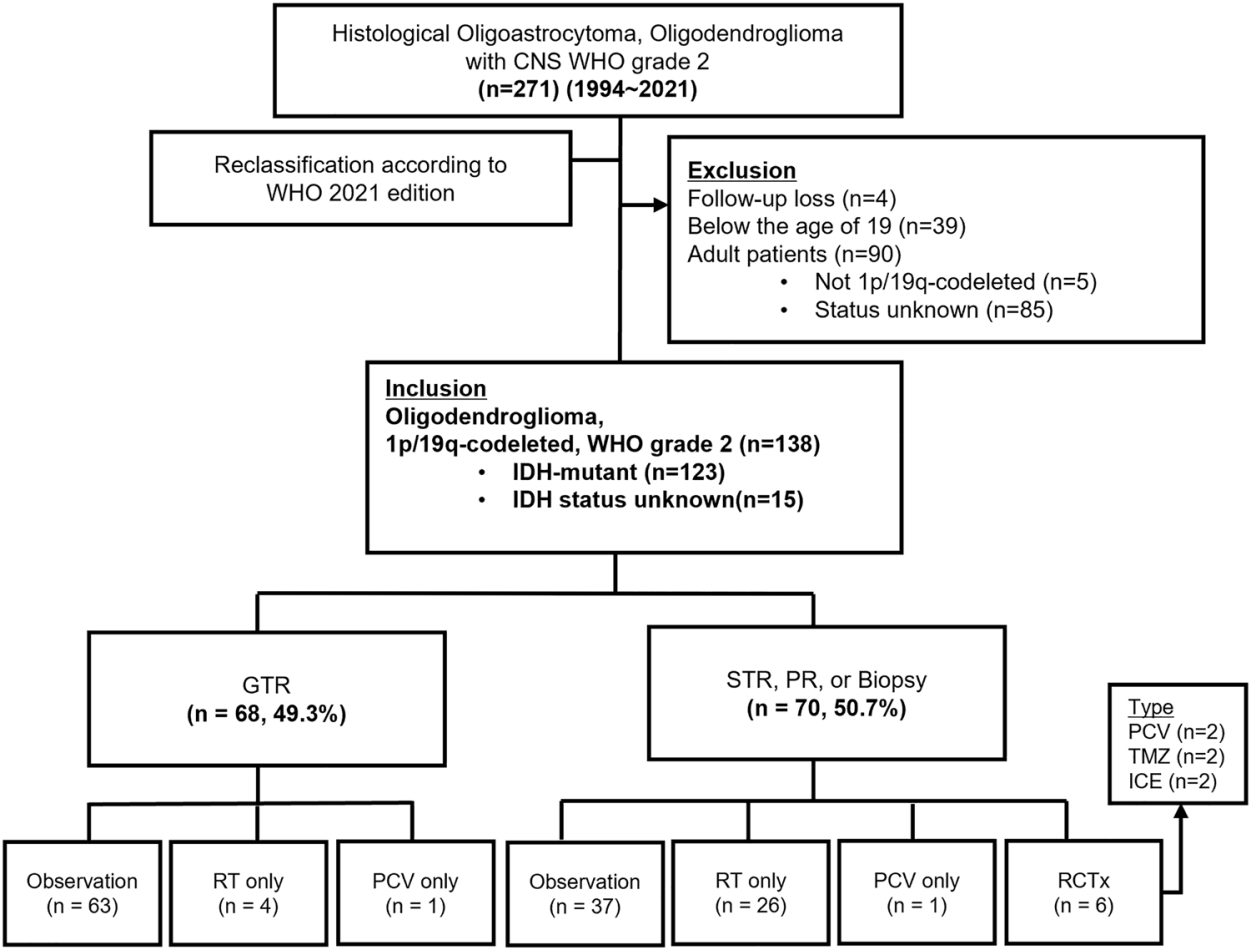
Flow chart of patient selection and treatment flow of 138 patients with WHO grade 2 oligodendroglioma. *WHO* World Health Organization, *CNS* Central Nervous System, *GTR* gross total resection, *STR* subtotal resection, *PR* partial resection, *RT* radiotherapy, PCV: procarbazine, lomustine, and vincristine, *RCTx* radio-chemotherapy, *TMZ* temozolomide, *ICE* Ifosfamide, carboplatin, etoposide

We included patients aged 19 years or older with a minimum follow-up duration of 12 months. Thirty-nine patients under the age of 19 were excluded from the study. Ninety patients without 1p/19q co-deletion or with unknown status were also excluded; among these, 5 patients did not have 1p/19q co-deletion, and 85 had unknown status. After excluding smaller cohorts, we ultimately included 138 patients who met the molecular criteria outlined in Fig. 1. The median follow-up duration for these patients was 6.6 years (range, 1.0–25.6).

### Histopathologic evaluation

All tumors were histopathological diagnosed as low grade oligodendrogliomas and molecularly defined according to the WHO classification. Our institute has determined the 1p/19q co-deletion status through fluorescence in situ hybridization (FISH) since 2008 and PCR detection prior to that. This test utilized Vysis (Abbott Laboratories, Chicago, IL) 1p36/1q25 and 19q13/19p13 FISH probes. A minimum of 60 tumor cells were counted, and a sample was deemed positive if at least 30% of tumor cells exhibited patterns consistent with 1p/19q deletion. Since 2012, our institute has routinely evaluated the IDH1 mutation status of glial tumors using immunohistochemistry (IDH1 R132H, MPQ-67, 1:50 dilution; Cell Marque, Rocklin, CA). For instances where IDH1 immunohistochemistry results were negative, subsequent DNA sequencing was conducted. For patients initially treated surgically before 2012, the molecular diagnosis was confirmed in additional surgeries performed due to tumor progression. Consequently, all cases were confirmed as 1p/19q co-deletion deletion, and 15 cases lacked data on IDH mutation status.

### Data collection

We retrospectively gathered clinical information, KPS scores, and pre- and post-surgery neurological symptoms from medical records. All patients underwent preoperative and postoperative magnetic resonance imaging (MRI) scans. Tumor characteristics, including location, contrast enhancement, and maximum diameter, were assessed through preoperative MRI scans.

The extent of resection (EOR) was assessed using postoperative MRI with reference to surgical reports. The EOR was determined by the absence of residual tumor volume on postoperative T2, or FLAIR MRI scans conducted within 48 h after surgery. EOR was categorized into: Gross Total Resection (GTR) indicating complete tumor removal; Subtotal Resection (STR) involving removal of 90% or more of the tumor with some remaining; Partial Resection (PR) with less than 90% removal of tumor, and biopsy where only a tissue sample is collected without substantial tumor removal.

Patients were divided into low-risk and high-risk groups based on RTOG risk criteria: low-risk for those ≤ 40 years with GTR, and high-risk for those > 40 years and/or having STR, PR, or biopsy [10, 13].

### Treatment and outcome assessment

We divided patients into two groups based on whether they received adjuvant treatment after surgical resection: an observation group and an adjuvant treatment group. The adjuvant treatment group was defined as those who received postoperative RTx, chemotherapy (CTx) or a combination of both (RCTx). OS and PFS were calculated from the time of tissue diagnosis following surgery until death or confirmed tumor progression by MRI.

### Statistical analysis

All statistical analyses in this study were conducted using R version 4.3.1 and R Studio software. In the descriptive statistics, categorical variables were represented using numbers and percentages, while continuous variables were represented using medians and ranges. OS, PFS, and their respective median times were calculated using the Kaplan–Meier method. The significance was assessed using the log-rank test. The Kaplan–Meier plots was generated using the survminer and ggplot2 packages in R.

Univariate and multivariable Cox regression analyses were conducted to identify potential risk factors affecting OS and PFS, with the multivariable analysis using backward elimination to select relevant variables. Age and tumor size were analyzed as continuous variables, while other variables are categorized into binary factors, including EOR (GTR or STR vs. PR or biopsy), and KPS score (KPS > 70 vs. ≤ 70). In our analysis, there was a correlation between tumor size and the EOR group (Spearman's rho = 0.486), but multicollinearity (VIF < 1.5) was not suspected when both variables were included in the model. Consequently, tumor size was excluded from the final multivariable model due to its lack of significance, as determined by backward elimination. A p-value < 0.05 was considered statistically significant, and hazard ratios (HR) with 95% confidence intervals (CI) were calculated. We applied the Least Absolute Shrinkage and Selection Operator (LASSO) regression to identify variables with non-zero coefficients associated with OS. To find an optimal λ (the degree of shrinkage), tenfold cross validation with minimum criteria was applied, where the final value of λ yielded minimum cross validation error. Remarkably, the selection of variables aligned with those identified through backward elimination (Supplementary Fig. 1).

## Results

### Demographic characteristics

The study included a total of 138 patients who were reclassified as 1p/19q co-deleted ODG WHO grade 2. In this study, men comprised 57.2% of the cohort, with a median age at diagnosis of 41.5 years (range, 19–79). Twenty-one percent of the patients were asymptomatic at the time of diagnosis, while seizures emerged as the most prevalent symptom, affecting 63% of patients. The median maximum tumor diameter measured 5 cm (range, 1.1–10.2). On preoperative MRI scans, contrast enhancement was observed in 43 individuals (31.2%), with the majority (69.6%) located in the frontal lobe.

Among these patients, 68 (49.3%) underwent GTR, while the remaining 70 (50.7%) underwent STR, PR, or biopsy (Fig. 1). Of those who underwent GTR, 63 (63/68, 92.6%) were observed, 4 (4/68, 5.9%) received RTx, and 1 received PCV CTx alone. In the STR, PR, or biopsy group, 37 patients (37/70, 52.9%) were observed, 26 patients (26/70, 37.1%) received RTx, and 6 (6/70, 8.6%) were treated with RCTx. Overall, within this cohort, 100 patients (72.5%) were observed after surgery, while 38 (27.5%) received adjuvant treatment, and only 4 cases were administered PCV (Table 1). Of the 36 patients who received postoperative RTx or RCTx, 5 reported experiencing mild memory impairment within 5 years of follow-up, primarily confirmed by the Korean version of the Mini-Mental State Examination (K-MMSE).

**Table 1:** Demographic characteristics

Variables	Total (n = 138^a^)
Sex	
Female	59 (42.8%)
Male	79 (57.2%)
Age	41.5 (19–79)
Preoperative symptoms	
Asymptomatic	29 (21.0%)
Seizure	87 (63.0%)
Headache/Dizziness	10 (7.2%)
Motor or Sensory deficit	6 (4.3%)
Cognitive impairment	5 (3.6%)
Motor aphasia	1 (0.7%)
Preoperative KPS score	
100	39 (28.3%)
90	36 (26.1%)
80	37 (26.8%)
70	23 (16.7%)
< 70	3 (2.2%)
Maximum tumor diameter (cm)	5 (1.1–10.2)
Small (< 5 cm)	65 (47.1%)
Large (≥ 5 cm)	73 (52.9%)
MRI findings	
No enhancement	95 (68.8%)
Enhancement	43 (31.2%)
Tumor Location	
Frontal	96 (69.6%)
Parietal	5 (3.6%)
Temporal	10 (7.2%)
Insular/BG	2 (1.4%)
Multiple	25 (18.1%)
IDH status	
IDH-mutant	123 (89.1%)
IDH status unknown	15 (10.9%)
RTOG risk	
High	105 (76.1%)
Low	33 (23.9%)
Extent of resection	
GTR	68 (49.3%)
STR	34 (24.6%)
PR	30 (21.7%)
Biopsy	6 (4.3%)
Postoperative management	
Observation	100 (72.5%)
Adjuvant therapy	38 (27.5%)
Radiotherapy	30 (21.7%)
Chemotherapy (PCV only)	2 (1.4%)
Radio-chemotherapy	6 (4.3%)
Regimen	
PCV	2 (1.4%)
TMZ	2 (1.4%)
ICE	2 (1.4%)
Treatment at progression	
Surgery only	14 (10.1%)
Surgery plus salvage therapy	24 (17.4%)
Radiotherapy	11 (8.0%)
Chemotherapy	10 (7.2%)
Radio-chemotherapy	7 (5.1%)
Others	3 (2.2%)
Survival outcome	
OS	
5 years	95.50%
10 years	76.10%
PFS	
5 years	60.10%
10 years	27.40%

*KPS* Karnofsky Performance Scale, *RTOG* Radiation Therapy Oncology Group, *GTR* gross total resection, *STR* subtotal resection, *PCV* procarbazine, lomustine, and vincristine, *TMZ* temozolomide, *ICE* Ifosfamide, carboplatin, etoposide, *OS* overall survival, *PFS* progression-free survival

^a^N (%); Median (range)

### Survival outcomes

The median PFS for patients in this study was 6.8 years, and the median OS reached 18.4 years. The 5-year and 10-year PFS rates were 60% and 27.4%, respectively. The 5-year and 10-year OS rates were 95.5% and 76.1%, respectively.

**Fig. 2:**
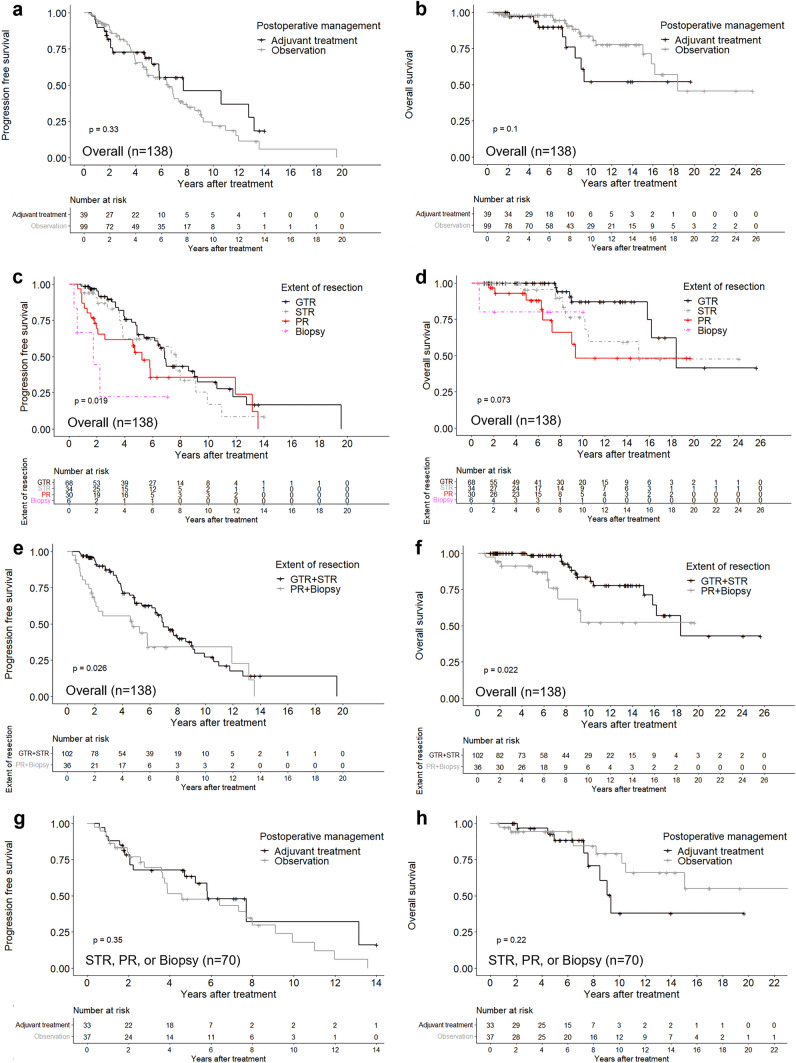
Kaplan–Meier curves for PFS and OS for all cases: **a**, **b** comparing postoperative observation with adjuvant treatment; **c**, **d** based on four different extent of resection groups; **e**, **f** based on two extent of resection groups (GTR or STR versus PR or biopsy); **g**, **h** for STR, PR, or biopsy groups, comparing postoperative observation with adjuvant treatment. p-value with statistical significance (p<0.05). *EOR* extent of resection, *GTR* gross total resection, *STR* subtotal resection, *PR* partial resection

Figure 2 presents Kaplan–Meier curves that show differences in PFS and OS among various treatment groups. There was no significant difference in PFS and OS between patients who underwent postoperative adjuvant treatment and those who were observed without treatment. However, the Kaplan–Meier curves for the four EOR groups showed a notable association with PFS (p = 0.019), but not OS. In addition, statistically significant improvements in both PFS and OS (p = 0.026 and p = 0.022, respectively) were noted in the GTR or STR group with less than 10% residual tumor when compared to the PR or biopsy group. Further analysis of the STR, PR, or biopsy group (n = 70), comparing postoperative adjuvant treatment to observation, did not reveal statistically significant differences in either PFS or OS.

### Prognostic factors for OS and PFS

Table 2 summarizes the results of univariate and multivariate analyses for prognostic factors influencing PFS and OS. Both univariate and multivariate analyses found that the presence of contrast enhancement (p < 0.001; HR: 2.36; CI: 1.46–3.81) and the PR or biopsy group (p = 0.022; HR: 1.8; CI:1.09–2.97) were predictors of worse PFS.

**Table 2:** Univariate and multivariate analysis of PFS and OS in patients with WHO grade 2 ODG

Variables^†^	PFS				OS			
	Univariate		Multivariate		Univariate		Multivariate	
	HR (95% CI)^﻿‡^	p-value	HR (95% CI)^‡^	p-value	HR (95% CI)^﻿‡^	p-value	HR (95% CI)^﻿‡^	p-value
Age	1 (0.98–1.03)	0.880			1.01 (0.96–1.05)	0.773		
Sex								
Male	1.32 (0.81–2.14)	0.259			1.05 (0.44–2.48)	0.914		
Seizure	1.07 (0.65–1.78)	0.787			0.35 (0.15–0.81)	**0.014**^‡$^	0.28 (0.11–0.7)	**0.006**^$^
KPS								
≤ 70	1.41 (0.83–2.38)	0.201			3.02 (1.24–7.35)	**0.015**^$^	2.32 (0.89–6.04)	0.085
Tumor size (cm)	1.12 (0.98–1.27)	0.103			1.45 (1.13–1.87)	**0.004**^$^		
MRI								
No enhancement	1 (ref.)		1 (ref.)		1 (ref.)		1 (ref.)	
Enhancement	2.31 (1.44–3.71)	**0.001**^$^	2.36 (1.46–3.81)	** < 0.001**^$^	7.53 (2.82–20.06)	** < 0.001**^$^	5.89 (2.16–16.07)	**0.001**^$^
EOR								
GTR	1 (ref.)	0.031^**‡**^			1 (ref.)	0.098		
STR	1.21 (0.68–2.16)	0.508			2.19 (0.77–6.25)	0.143		
PR	1.66 (0.94–2.93)	0.082			3.50 (1.26–9.71)	0.016^‡^		
Biopsy	4.22 (1.48–12.05)	0.007^**‡**^			4.08 (0.49–33.94)	0.193		
EOR (2 groups)								
GTR or STR	1 (ref.)		1 (ref.)		1 (ref.)		1 (ref.)	
PR or Biopsy	1.74 (1.06–2.87)	**0.026**^$^	1.8 (1.09–2.97)	**0.022**^$^	2.59 (1.11–6.04)	**0.022**^$^	2.78 (1.09–7.05)	**0.032**^$^
RTOG risk								
High	1 (ref.)				1 (ref.)			
Low	1.22 (0.73–2.06)	0.445			1.74 (0.67–4.48)	0.254		
Postoperative management								
Adjuvant Tx	1 (ref.)				1 (ref.)			
Observation	1.3 (0.76–2.23)	0.332			0.5 (0.21–1.18)	0.113		

*PFS* progression-free survival, *OS* overall survival, *KPS* Karnofsky Performance Scale, *MRI* magnetic resonance imaging, *EOR* extent of resection, *GTR* gross total resection; STR = subtotal resection; *PR* partial resection, *RTOG* Radiation Therapy Oncology Group; *Tx* treatment

^†^Age and tumor size were analyzed as continuous variables, while the other variables were analyzed as categorical variables

^‡^*HR* Hazard Ratio, *CI* Confidence Interval

^$^p-value with statistical significance (p<0.05)

Transitioning from the analysis of PFS to OS, similar variables were scrutinized for their impact on patient outcomes. In the univariate analysis, seizure, KPS score, tumor size, contrast enhancement, and the EOR group were prognostic factors for OS (p = 0.014, 0.015, 0.004, < 0.001, and 0.022, respectively). Multivariate analysis revealed seizures at presentation as a favorable prognostic factor for OS (p = 0.006; HR: 0.28; CI: 0.11–0.7). Conversely, contrast enhancement on MRI (p = 0.001; HR: 5.89; CI: 2.16–16.07) and the PR or biopsy group (p = 0.032; HR: 2.78; CI: 1.09–7.05) were associated with worse OS outcomes.

## Discussion

This study presents the largest patient cohort analysis on 1p19q-codeleted ODG WHO grade 2 conducted by a single institution to date, providing a comprehensive analysis of long-term outcomes and prognostic factors for survival. As detailed in Table 3, the observed median PFS of 6.8 years and median OS of 18.4 years in our cohort are consistent with the favorable prognosis associated with this molecular subtype, consistent with previous studies [6, 14–17]. Notably, these results are better compared to the 24.7 months of median PFS, and 50.8 months of median OS reported in our recent study involving 95 patients with anaplastic ODG at our institution [18]. Our previous research showing the impact of the EOR on the prognosis of anaplastic ODG is consistent with findings from other studies [18–20]. However, the influence of EOR on survival in WHO grade 2 ODG remains a subject of debate.

**Table 3:** Comprehensive review of studies on lower grade ODG

Authors & Year	Dx	No. of Pts	Period	Median F/U period (yrs)	Seizure (%)*	EOR (%)		Postop Adjuvant Tx	OS (%)	Median OS (yrs)	PFS (%)	Prognostic factor
						GTR	Non-GTR					
El-Hateer et al. [14], 2009	ODG	38 (55%)	1992–2006	6.1	78	27	72	RTx (21.7%), CTx (8.6%)	5 yr: 83 10 yr: 63	6.5 (High risk), 12.8 (Low risk)	5 yr: 46 10 yr: 7.7	Seizure, Contrast enhancement on MRI
Franceschi et al. [15], 2019	ODG	93	NA	8	NA	25.8	74.2	RTx (19.4%), CTx (18.3%), RCTx (8.6%)	NA	18	NA	Any type of postsurgical treatment
Kavouridis et al. [6], 2019	ODG	140	NA	4.7	57.1	42.8	52.8	RTx (2.8%), CTx (19.3%), RCTx (20%)	5 yr: 96.9 10 yr: 84.1	NA	5 yr: 38.5 10 yr: 24.1	Postoperative residual volume
Mair et al. [16], 2022	ODG grade2	131	1992–2019	NA	63.7	33.6	65.6	early postop Tx (13.3%)	NA	15.2 (Early Tx), 16.7 (No Tx)	NA	Extent of resection
Carstam et al. [17], 2023	ODG grade2	126	1998–2016	12	73.4	77	23	RTx (35.7%), CTx (19.0%), RCTx (7.9%)	NA	17.8	NA	Age, Tumor diameter, Functional status (KPS < 80)
Hervey-Jumper, S. L. et al. ^[22]^, 2023	ODG grade2	190	1998–2017	11.7	80.7	61	39	RTx (37.9%), CTx (47.1%),	NA	NA	Median PFS: 11.7	Extent of resection, Postoperative residual volume
Present study	ODG grade2	138	1994–2021	6.5	63	49.3	50.7	RTx (21.7%), CTx (1.4%), RCTx (4.3%)	5 yr: 95.5 10 yr:76.1	18.4	5 yr: 60.1 10 yr: 27.4	Seizure, Extent of resection, Contrast enhancement on MRI

*Dx*  diagnosis, *Pts* patients, *F/U* follow-up, *yrs* years, *EOR* extent of resection, *GTR* gross total resection, *STR* subtotal resection; *Tx * treatment; *OS* overall survival, *PFS* progression-free survival, *ODG* oligodendroglioma, *NA* not applicable; *RTx* radiotherapy; CTx = chemotherapy; *RCTx* Radio-chemotherapy, *MRI* magnetic resonance imaging, *KPS* Karnofsky Performance Scale

*The values displayed indicate the percentage reported in each study

Numerous studies have concluded that a more extensive EOR significantly impacts survival, even when considering the molecular characteristic of 1p/19q co-deletion [6, 19, 21–23]. Our findings distinctly highlight that the GTR or STR group, with less than 10% residual tumor, showed better PFS and OS compared to the PR or biopsy group with higher residual tumor levels. This suggests that smaller residual tumor volumes are associated with improved outcomes. In a recent study by Hervey-Jumper, S. L. et al.[22], the evaluation of volumetric EOR revealed that ODG patients with postoperative tumor volume of 4.6 mL or less had the longest OS, which aligns with our findings. Moreover, in patients with LGG, achieving an EOR of 75% or higher improved OS, while an EOR of 80% or higher improved PFS[22]. In contrast to studies indicating that the EOR influences survival outcomes, according to the findings studied by Carstam et al., it was reported that initial surgical strategies do not affect survival outcomes. However, this study identifies limitations in accurately evaluating the EOR due to the characteristics of a multicenter study, as well as the absence of postoperative MRIs in the initial study participants [17]. Additionally, a recent study reported that while the EOR impacts PFS, it does not affect OS [16]. In this study, it is reported that 19% of patients received adjuvant CTx within 6 months after surgery, and 35.7% received RTx. When including the percentages of patients who received CTx and RTx up until the point of disease progression, the totals increase to 63.5% and 71.4%, respectively. This suggests that ODG may be more sensitive to adjuvant treatment compared to other subtypes of glioma, potentially diminishing the influence of EOR on overall survival [24]. In our patient cohort, 72.5% underwent watchful observation without additional treatment after surgery, which likely allowed for a more accurate assessment of the role of the EOR.

While ongoing discussions persist regarding the impact of early postoperative treatment on OS in patients with WHO grade 2 ODG [15, 16, 25], recent comprehensive clinical studies have demonstrated that adding PCV CTx to RTx not only improves PFS but also extends OS in patients at high risk [7, 10]. In our study, paradoxically, patients who received RTx or CTx after undergoing STR, PR, or biopsy exhibited no differences in PFS and OS compared to those who were simply observed after surgery. Although the current policy at our institution is to administer PCV CTx after RTx in high-risk groups based on the RTOG risk criteria, in many patients included in this study, RTx or CTx was selectively performed in patients with a poor prognosis based on radiological findings. In particular, the PCV CTx was restricted to a few cases primarily due to concerns about adverse effects, such as neutropenia, highlighting a significant limitation of our study.

Our multivariate analysis revealed that contrast enhancement on preoperative MRI as an unfavorable prognostic factor for both PFS and OS. Previous studies have reported contrast enhancement in 25 to 56% of patients with lower grade ODG [14, 17, 26–28], a range comparable to the 31.2% observed in our study. Several studies have also identified contrast enhancement on MRI scans as a potential prognostic factor in IDH-mutant gliomas [14, 26, 29]. In contrast, some studies have reported no correlation between contrast enhancement and PFS or OS [16, 17]. A comparative study on IDH mutation status reveals that the presence of contrast enhancement in diffuse IDH wild-type gliomas had no significant impact on survival, whereas its presence is associated with a poorer prognosis in IDH-mutant gliomas [26]. Although often considered an indicator of aggressive tumor behavior, contrast enhancement may also reflect regions of anaplasia not sampled during biopsy. Ensuring accurate biopsy interpretation is crucial to avoid underestimating disease severity and to guide appropriate treatment strategies. To prevent the underestimation of focal enhancing tumors, using 5-aminolevulinic acid (5-ALA) guided tissue sampling can be advantageous. This technique enhances the precision of biopsy sampling by making tumor cell visibility under fluorescence, thereby improving diagnostic accuracy [30].

Seizures represent the most common symptom in patients with LGGs. In glioblastoma, seizures occur in approximately 40–60% of cases, whereas in oligodendroglioma, the incidence rises to 70–90%, with seizures often being the initial symptom in about 60% of cases [31]. In our study, seizures were the initial symptom in approximately 63% of the cases. Previous studies have confirmed that experiencing a single seizure before surgery is a positive survival indicator for patients with LGG. Conversely, relapsing seizures or an escalation in epileptic activity is predictive of anaplastic transformation [14, 29, 31, 32]. Our multivariate analysis identified the presence of seizures at presentation as a favorable prognostic factor for OS, which may be attributed to an earlier diagnosis and potentially less aggressive tumor behavior in these patients.

## Strengths and limitations

While our study contributes valuable insights into the long-term outcomes and prognostic factors for patients with this rare tumor subtype, it is crucial to acknowledge the inherent limitations associated with a retrospective, single-institution study design. Furthermore, the relatively short median follow-up period of 6.6 years is an additional limitation, given the long median survival of the cohort studied. Additionally, this study included 15 patients (10.9%) with unclear IDH mutation status, which poses its own set of limitations. Although IDH wild-type, 1p/19q co-deleted gliomas have been documented [33], they are rare and are not classified in the latest WHO classification. Furthermore, the prognosis of patients with unknown IDH status in this study was similar to that of those with low-grade tumors. One notable characteristic of our study was the heterogeneity in managing patients with ODG WHO grade 2, particularly a significant number of patients were observed without adjuvant treatment. In the context of well-established standard treatments like PCV CTx and the recently FDA approved IDH mutant inhibitor ‘Vorasidenib’ through Phase III trials [34], understanding the impact of the EOR on the disease prognosis becomes increasingly challenging. Against this backdrop, we believe our cohort presents a valuable opportunity to explore how EOR alone influences the outcome of ODG WHO grade 2. Following the introduction of IDH mutant inhibitors, future research should focus on optimizing the EOR to preserve functional outcomes and determining the optimal timing for surgical intervention. Advancements in molecular profiling and imaging techniques promise to deepen our understanding of the biological characteristics of these tumors, potentially leading to more precisely tailored and effective management approaches.

## Conclusions

We observed favorable long-term outcomes and identified crucial prognostic factors. These include the initial seizure presentation and the absence of contrast enhancement on MRI, both of which significantly impact survival outcomes. Our findings emphasize the significance of the EOR as a crucial prognostic factor in improving PFS and OS in patients with WHO grade 2 ODG similar to the findings from our previous study involving 95 patients with anaplastic ODG [18].

### Supplementary Information

Supplementary Material 1.Fig. 1 Least absolute shrinkage and selection operator (LASSO) regression (a) with tenfold cross-validation (b) to reduce the dimensionality of the grouping features in overall survival. The minimum error was found to correspond to four features: MRI enhancement, EOR (in 2 groups), KPS score, and seizure.

## Data Availability

The datasets generated during and/or analyzed during the current study are available from the corresponding author on reasonable request.
