# Supplementary figures and images for: Long-term outcomes of CNS WHO grade 2 oligodendroglioma in adult patients: a single-institution experience
Source: Discov Oncol. 2024 Jul 6;15:268. doi: 10.1007/s12672-024-01136-4 (PMC11227491; doi:10.1007/s12672-024-01136-4)

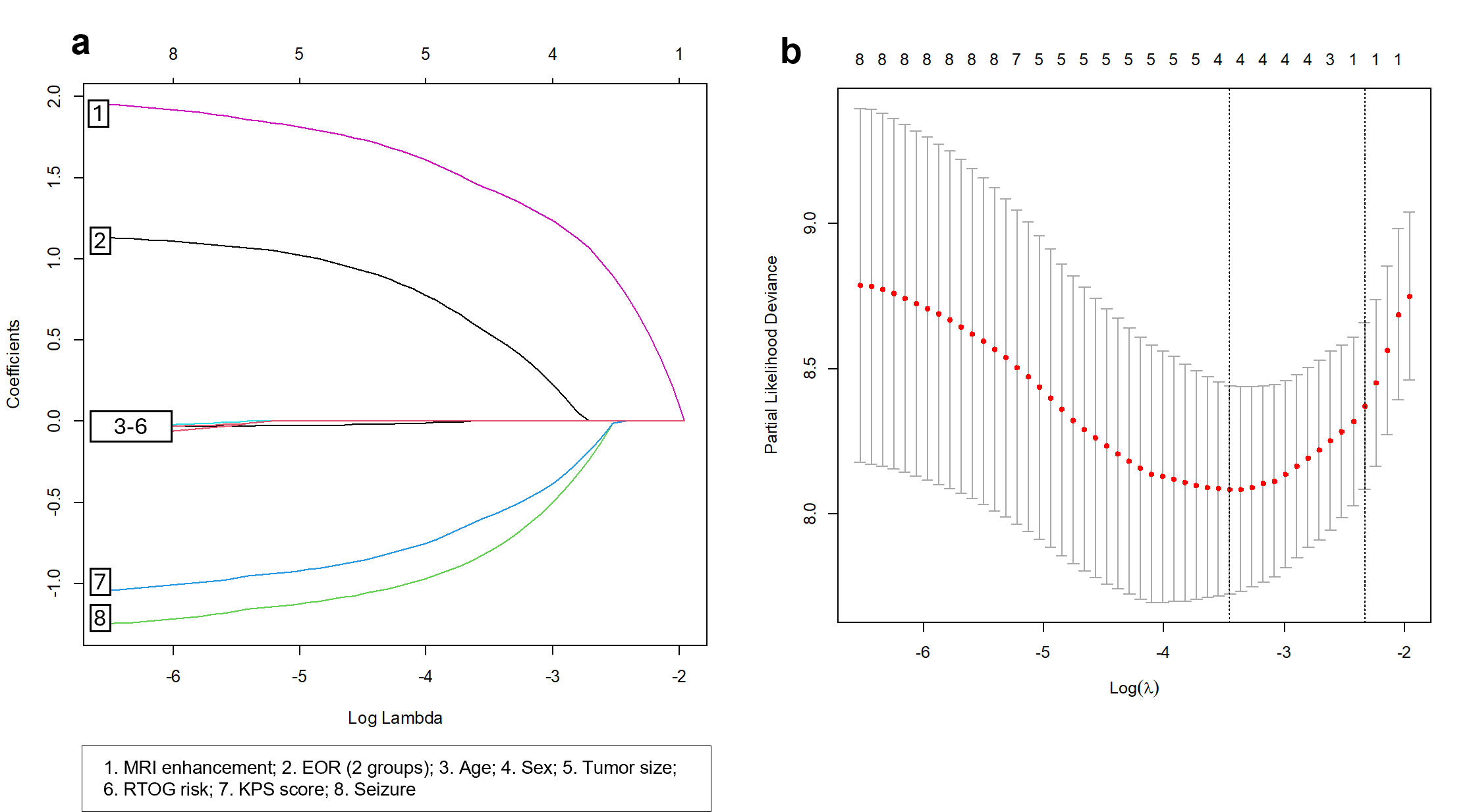

Supplement: Supplementary file 1 — Supplementary Material 1.Fig. 1 Least absolute shrinkage and selection operator (LASSO) regression (a) with tenfold cross-validation (b) to reduce the dimensionality of the grouping features in overall survival. The minimum error was found to correspond to four features: MRI enhancement, EOR (in 2 groups), KPS score, and seizure. [file 12672_2024_1136_MOESM1_ESM.tif]
